# A multi-component stair climbing promotional campaign targeting calorific expenditure for worksites; a quasi-experimental study testing effects on behaviour, attitude and intention

**DOI:** 10.1186/1471-2458-12-423

**Published:** 2012-06-11

**Authors:** Frank F Eves, Oliver J Webb, Carl Griffin, Jackie Chambers

**Affiliations:** 1 School of Sport and Exercise Sciences, University of Birmingham, Edgbaston, Birmingham, B15 2TT, UK; 2 School of Sport, Exercise and Health Sciences, Loughborough University, Leicestershire, LE11 3TU, UK; 3 Heart of Birmingham Teaching Primary Care Trust NHS, Birmingham, B16 9PA, UK

## Abstract

****Background**:**

Accumulation of lifestyle physical activity is a current aim of health promotion, with increased stair climbing one public health target. While the workplace provides an opportunity for regular stair climbing, evidence for effectiveness of point-of-choice interventions is equivocal. This paper reports a new approach to worksite interventions, aimed at changing attitudes and, hence, behaviour.

****Methods**:**

Pre-testing of calorific expenditure messages used structured interviews with members of the public (n = 300). Effects of multi-component campaigns on stair climbing were tested with quasi-experimental, interrupted time-series designs. In one worksite, a main campaign poster outlining the amount of calorific expenditure obtainable from stair climbing and a conventional point-of-choice prompt were used (Poster alone site). In a second worksite, additional messages in the stairwell about calorific expenditure reinforced the main campaign (Poster + Stairwell messages site). The outcome variables were automated observations of stair and lift ascent (28,854) and descent (29,352) at baseline and for three weeks after the intervention was installed. Post-intervention questionnaires for employees at the worksites assessed responses to the campaign (n = 253). Analyses employed Analysis of Variance with follow-up Bonferroni t-tests (message pre-testing), logistic regression of stair ascent and descent (campaign testing), and Bonferroni t-tests and multiple regression (follow-up questionnaire).

****Results**:**

Pre-testing of messages based on calorific expenditure suggested they could motivate stair climbing if believed. The new campaign increased stair climbing, with greater effects at the Poster + Stairwell messages site (OR = 1.52, 95% CI = 1.40-1.66) than Posters alone (OR = 1.24, 95% CI = 1.15-1.34). Follow-up revealed higher agreement with two statements about calorific outcomes of stair climbing in the site where they were installed in the stairwell, suggesting more positive attitudes resulted from the intervention. Future intentions for stair use were predicted by motivation by the campaign and beliefs that stair climbing would help weight control.

****Conclusions**:**

Multi-component campaigns that target attitudes and intentions may substantially increase stair climbing at work.

## **Background**

Insufficient physical activity is a major problem [1]. In 2008, 61% of men and 71% of women in England reported insufficient activity, with objective measurements revealing 94% of men and 96% of women below recommended levels [2]. Accumulating activity during daily life is one public health approach to this problem [3,4]; walking rather than driving and choosing stairs rather than lifts would increase lifestyle physical activity.

Stair climbing uses 9.6 times the energy of the resting state [5], i.e., requires more energy per minute than jogging or rowing. As ascent requires two to three times the energy expenditure of descent, increased stair climbing is the preferred public health goal [5,6]. To increase stair use, messages are positioned at the ‘point-of-choice’ between stairs and the escalator, encouraging individuals to climb the stairs for their health. For a choice between stairs and escalators in public access settings, stair climbing increases on average +5.9%. When pedestrians choose between stairs and a lift in worksites, however, the average increase for stair use is +0.1% [7-11]. This equivocal evidence in worksites is problematic; regular stair climbing provides the greatest dividend and worksites are a plausible location for its occurrence. In this paper, we use the theoretical mechanisms underlying stair climbing interventions to provide a new approach for worksites.

One influential model of health behaviour, the Theory of Planned Behaviour TPB; [12-14], posits that behaviour is determined by intentions to perform it. Attitudes, the strongest predictor of intentions to be physically active, reflect positive and negative beliefs about the consequences of that behaviour [14]. For example, beliefs that stair use will influence weight have been related to observed stair climbing [15]. Mass media campaigns target attitudes by providing information about the positive benefits of health enhancing behaviour and/or the negative costs of health threats. Hence, they target motivations towards the behaviour by providing the reason *why* the behaviour should be performed. Mass media campaigns target decisions to enhance one’s health, i.e. intentions, by outlining future benefits or costs. By contrast, a second type of intervention helps translate intentions into action, e.g., making an implementation intention [15] or planning participation in the intended behaviour [16]. Point-of-choice prompts for stair climbing are a further example of these volitional interventions. Exposure to a prompt is fleeting, perhaps lasting a few seconds during an individual’s journey. This brief exposure provides insufficient time to change attitudes. Rather, point-of-choice prompts alert individuals with *existing* intentions to be physically active that stair climbing is a health enhancing means of meeting their intention. Thus, point-of-choice prompts help translate *prior* intentions to be more physically active into action. Hence, a campaign that targets attitudes, the precursors of intentions, in addition to prompting the behaviour at the point-of-choice, may optimize the impact on stair climbing. As adults spend half their waking hours at work [17], the workplace allows repeated exposure of individuals to the more elaborate campaign required to change attitudes.

The campaign that we report added two elements that targeted attitudes to a conventional point-of-choice campaign. First, an extended message translated information about the calorific expenditure of stair climbing into lay terms. The core message informed employees that ‘One flight uses about 2.8 calories but 10 flights a day would use 28 calories. Over a year that adds up to 10,000+ calories; that’s more than four days worth of food’. This core message specified the *amount* of stair climbing required to achieve the outcome see [18]. It aimed to contrast an achievable daily task, i.e., 10 flights, with the benefit accumulated over a year to maximize the possible gain, i.e., four days without food. The main text in one worksite (Poster alone) was compared with a second worksite (Poster + Stairwell messages) in which supplementary messages in the stairwell described calorific outcomes of stair use, e.g., ‘burns more calories per minute then jogging’. These supplementary messages also target attitudes. Thus, the extended text and supplementary messages targeted attitudinal change, whereas the conventional point-of-choice prompt at the lift button aimed to translate any changed intentions into action.

### Overview of research sections of the paper

**Figure 1:**
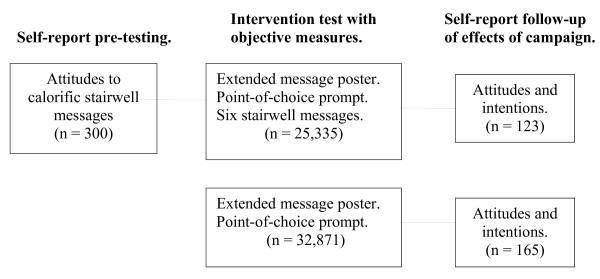
Schematic of the evidence presented in the paper and the intervention components at the two worksites.

The schematic in Figure 1 summarises the evidence presented in the paper. First, we pre-tested calorific expenditure messages for use as supplementary messages for the stairwell; weight related messages were not reported as motivating in previous pre-testing [19]. Next, an observational study used two worksites to test the new campaign aimed at attitudinal change on stair climbing. Both worksites displayed the extended message and conventional point-of-choice prompts. For one worksite, Poster + Stairwell messages, the supplementary messages were added in the stairwell. We predicted an increase in stair usage when the campaign was installed, with a greater increase at the Posters + Stairwell messages site than the Poster alone one. Finally, a post-campaign questionnaire tested the effects of the campaign on individuals’ attitudes and intentions towards stair use. 

## **Methods**

Ethical approval for this study was obtained from the Ethics Subcommittee of the School of Sport and Exercise Sciences, University of Birmingham.

### Message pre-testing

Two messages that contained specific consequences based on calorific expenditure were included in a large interview study of potential stair climbing messages. Pre-testing used a replicated cohorts design to reduce the influence of sampling variability. This design interviews two separate cohorts and restricts conclusions only to results replicated across the cohorts see [20]. Members of the public in two cohorts (2 x n = 150) were interviewed by postgraduate students beside a 6-floor building in the West Midlands, UK. Participants read the stem *‘*Regular stair climbing’ and the statements ‘burns more calories per minute then jogging’ and ‘burns more calories per minute than rowing’. First, respondents rated how much they agreed with each message on a five point scale with the verbal labels ‘1 = not at all’, ‘2 = a little’, ‘3 = moderately’, ‘4 = a lot’ and ‘5 = very much’. Using the same scale, respondents then rated how much ‘each message would encourage them to use the stairs’ (motivation rating). To simulate effects of endorsement by health promotion agencies, half the interviewees in each cohort were told that these calorific consequences of stair climbing were true (Told true). Thus, before giving motivation ratings, respondents in the Told true condition were informed that all the messages were true. We predicted higher motivation ratings, relative to agreement ratings, in the Told true groups.

### Worksite interventions

#### Location

Two worksites in the centre of a large city in the West Midlands (UK) were recruited (Posters alone; City Council building, 1200 employees, five floors: Posters + Stairwell messages; Water Supply company, 500 employees, four floors). The allocation of campaigns (Posters alone vs. Posters + Stairwell messages) between the sites was determined by a coin toss. Both sites had stairs and a lift positioned in the foyer and involved predominantly white collar workers. Despite these similarities, the stairs and single lift at the Posters + Stairwell messages site were adjacent, whilst at the Posters alone site the stairs and a pair of lifts were separated by about 4 meters. As more available lifts are likely to reduce stair climbing [7], one could expect lower baseline rates of stair usage at the Poster alone site.

#### Intervention materials

**Figure 2:**
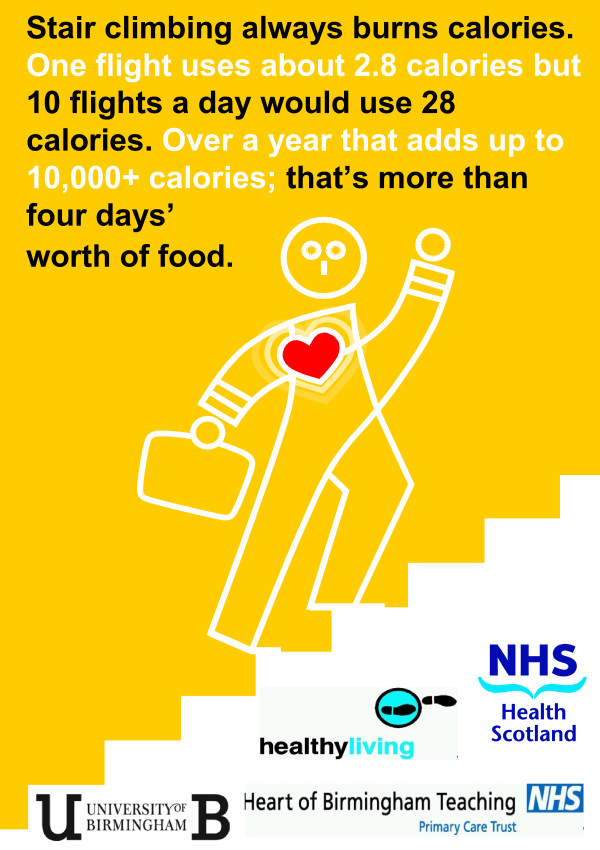
The A2 core message campaign poster.

**Figure 3:**
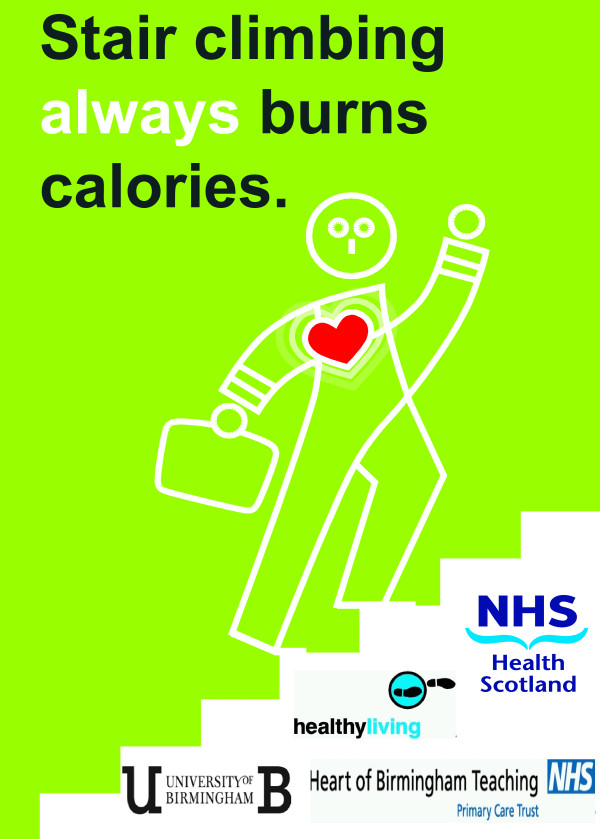
The A4 point-of-choice prompt poster.

The core campaign was composed of two components. The extended message targeting attitude, namely ‘Stair climbing always burns calories. One flight uses about 2.8 calories but 10 flights a day would use 28 calories. Over a year that adds up to 10,000+ calories; that’s more than four days worth of food’, was displayed on A2 posters that depicted a manikin climbing stairs (Figure 2). One poster was positioned in the foyer and one halfway up each flight of stairs. Each poster message was endorsed prominently by the highly credible sources of the Heart of Birmingham Teaching NHS Primary Care Trust, Healthy Living, NHS Health Scotland and the University of Birmingham. An arrow at the lift button pointed to the stairs with the message ‘Stairs this way’. Above this arrow, an A4 poster displayed the same manikin as the main poster and a conventional point-of-choice prompt, ‘Stair climbing always burns calories’ (Figure 3). For the Posters + Stairwell messages site, six different messages on blue backgrounds were positioned on the wall beside the stair risers between each floor. The messages were ‘Regular stair climbing helps control your weight’, and five messages beginning with the stem *‘*Stair climbing’ and using the five different roots of ‘always burns calories’, ‘burns more calories per minute than jogging’, ‘burns more calories per minute than rowing’, ‘is free exercise’ and ‘provides daily exercise’.

#### Measurement of stair and lift usage

Employees entering and exiting the ground floor lift(s) and stairwell were recorded by unobtrusive automatic counters. One set of counters monitored the stairwell and the other set the lift(s). These counters used two infrared beams in the horizontal plane and purpose built circuitry to distinguish the order in which the beams were broken. Thus, entry could be distinguished from exit for the lift(s) and the stairwell. The output of this circuitry was stored on data loggers (μlogger RVIP, Zeta-tec, England), one each for entry and exit, which counted the number of pulses per minute. Thus, they were able to distinguish ascending and descending stair users, and ingress (ascent) and egress (descent) for the lift. Correlations of the automatic counts.min^-1^ with direct observations for entering and exiting the stairs made on two occasions were r(249) = .94 and r(249) = .95, respectively, with equivalent correlations for entering (r (321) = .93) and exiting (r (321) = .94) the lifts (all p < .001). While data were recorded for 24 hours each day, only data from 7:00 am to 5:59 pm were included in analyses. After a 1-week baseline period, the stair climbing campaign was introduced and recording continued for another three weeks.

#### Follow-up Questionnaire

Two weeks after the end of monitoring, a follow-up questionnaire was distributed through the internal mail. Participants responded to all questions on 6-point scales with the anchors strongly disagree (1) to strongly agree (6). Two items assessed motivation by the campaign, ‘The message on the poster encouraged me to use the stairs’ and ‘I found the poster in the lobby encouraged me to use the stairs’ (Cronbach’s α = 0.86). Intentions to use the stairs more *in the future* were assessed with the items ‘I will try to use the stairs more than I used to’ and ‘I intend to use the stairs more than I used to’ (Cronbach’s α = 0.87). Respondents rated agreement with four statements displayed in the stairwell at the Poster + Stairwell messages site (‘Stair climbing provides daily exercise’, ‘Regular stair climbing helps control my weight’ ‘Stair climbing burns more calories per minute than jogging’, ‘Stair climbing burns more calories per minute than rowing’) and four comparison statements not used in the campaign. These contained the stem ‘Regular stair climbing’ and the roots ‘will keep me fit’, ‘will reduce my risk of a heart attack’, ‘can reduce my cholesterol levels’ and ‘will protect me from osteoporosis (brittle bones)’. We predicted higher agreement with the statements positioned in the stairwell of the Posters + Stairwell messages site relative to the Poster alone one and no differences between the two sites for messages not positioned at either site.

### Analysis

Interview data were analysed with a 2 x 2 mixed model ANOVA with the between subject factor of Group (Told true vs. Told nothing) and the within subject factor of Rating (Agreement vs. Motivation). Follow-up tests used Bonferroni corrected t-tests. Stair counts were analysed with logistic regression with the potential predictors of worksite, intervention, and pedestrian traffic. The follow-up questionnaire was analysed with Bonferroni corrected t-tests and step-wise multiple regression.

## **Results**

### Message pre-testing

The Told true group here aimed to mimic effects of endorsement by health promotion agencies. All analyses in the 2 x 2 mixed model ANOVAs contained the predicted interaction of Group and Rating (all p < .01), reflecting an increase in motivation ratings for the Told true group c.f. [20]. Table 1a provides mean ratings (SE) for each message and summarizes the effects of post-hoc Bonferroni corrected t-tests. For purposes of comparison, Table 1b shows results for five different messages from the same interviews, the data for which were not presented previously for each individual message [20]. 

**Table 1:** Agreement and motivation ratings (SE) for messages at pre-testing

**a) Calorific expenditure messages**	**Told nothing**		**Told true**	
	**Agree**	**Motivate**	**Agree**	**Motivate**
Interview Cohort 1				
Burns more calories per minute than jogging	2.27	2.42	2.46	3.67***^a^
	(0.16)	(0.16)	(0.13)	(0.14)
Burns more calories per minute than rowing	1.69	1.99	1.81	3.74***
	(0.11)	(0.14)	(0.11)	(0.15)
Interview Cohort 2				
Burns more calories per minute than jogging	2.60	2.44	2.77	4.53***
	(0.15)	(0.17)	(0.13)	(0.08)
Burns more calories per minute than rowing	2.20	2.39	2.79	4.40***
	(0.15)	(0.17)	(0.12)	(0.09)
b) Comparison messages				
Aids weight loss	2.59	1.97†††^b^	2.64	3.33**
	(0.16)	(0.16)	(0.16)	(0.15)
Is daily exercise	3.87	3.34††	3.39	3.41
	(0.14)	(0.14)	(0.15)	(0.13)
Exercises your heart	4.01	3.35†††	3.64	3.99
	(0.13)	(0.16)	(0.08)	(0.13)
Helps to keep you healthy	3.30	2.69†††	3.54	3.63
	(0.16)	(0.15)	(0.14)	(0.15)
Keeps you fit	4.07	3.50††	3.49	3.59
	(0.12)	(0.14)	(0.12)	(0.14)

Table legend: ^a^ Motivate > Agreement: * = p < .05, ** = p < .01, *** = p < .001.

^b^ Agreement > Motivate: † = p < .05, †† = p < .01, ††† = p < .001.

Agreement ratings were low for all messages that concerned calorific expenditure or weight relative to the other messages, irrespective of whether the other messages were general (daily exercise) or outlined consequences (keep healthy, exercise heart, keep fit). Formal comparisons between the messages on agreement ratings confirmed this (all prob. <.001). For the group Told nothing, agreement ratings were greater than motivation ratings for *all* messages not involving calorific expenditure as reported previously [20]. Finally, informing respondents that the messages were true increased their apparent motivational properties. Hence, there were no differences between agreement and motivation ratings for messages not explicitly related to weight control. Further, motivation ratings were significantly greater than agreement ratings for all messages relevant to weight control, suggesting this type of message could be effective for stair climbing if endorsed by health promotion agencies.

### Effects of intervention

Preliminary inspection of the data with box-plots revealed that pedestrian traffic levels greater than 20 min^-1^ were outliers and any such time points were excluded from further analysis (2.9% of observations). Thus, 28,854 and 29,352 separate choices between the stairs and lifts remained for analysis of ascent and descent respectively.

**Figure 4:**
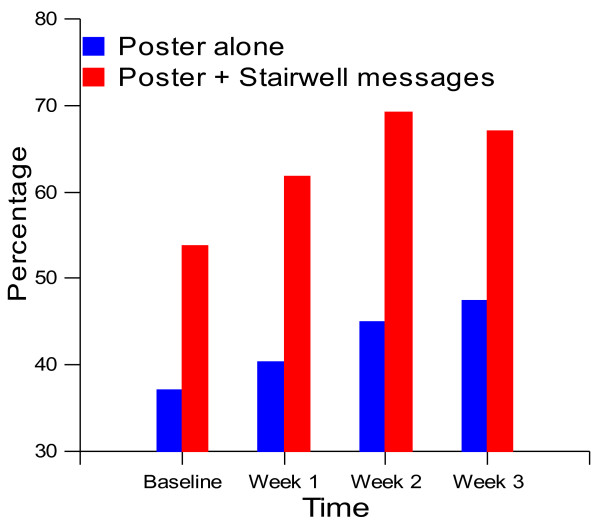
Stair climbing at baseline and during the intervention at the Poster + Stairwell Messages and Poster alone worksites.

Table 2 summarises the omnibus analyses. Significant effects of the intervention on stair climbing contrasted with no effect on stair descent. As the intervention effect on stair climbing interacted with the site, separate analyses were computed for each site (all p < .001 unless stated). There was a greater overall effect of the Poster + Stairwell messages campaign (+12.3%; Odds Ratio (OR) = 1.52, 95% Confidence Interval (CI) = 1.40-1.66) than with the Posters alone (+7.2%; OR = 1.24, CI = 1.15-1.34). Figure 4, which depicts the percentage of individuals climbing stairs at the two sites over successive weeks, suggests that incremental changes occurred. Follow-up analyses revealed an increase between baseline and week one (Poster + Stairwell messages: OR = 1.36, CI = 1.23-1.50; Poster alone: OR = 1.12, CI = 1.03-1.22, p = .007) and a further increment between weeks one and two (Poster + Stairwell messages: OR = 1.33, CI = 1.18-1.50; Poster alone: OR = 1.18, CI = 1.07-1.31). Any incremental effect had dissipated by week three (Poster + Stairwell messages: OR = 0.88, CI = 0.78-1.00, p = .04; Poster alone: OR = 0.98, CI = 0.88-1.08). For stair descent, Table 2 reveals no effects of the intervention, nor any interaction between the interventions and site (Poster + Stairwell messages; baseline 66.4%, intervention 64.2%; OR = 0.99, CI = 0.92-1.06: Poster alone; baseline 70.8%, intervention 71.6%; OR = 1.03, CI = 0.96-1.11).

**Table 2:** Summary of the overall effects of the campaigns on stair climbing and descent

**Variable**	**Stair climbing** **(n = 28 854)** **OR (95% CI)**	**Stair descent** **(n = 29 352)** **OR (95% CI)**
Intervention > baseline	1.24*** ^a^	1.03
	(1.15-1.34)	(0.96-1.11)
Interaction between intervention and worksite	1.23***	0.95
(Poster + Stairwell messages > Poster alone)	(1.10-1.38)	(0.86-1.05)
Overall effects of worksite	2.02***	0.80***
(Poster + Stairwell messages vs. Poster alone)	(1.83-2.23)	(0.74-0.87)
Effects of pedestrian traffic volume.min^-1^ (continuous variable)	0.95***	0.94***
	(0.95-0.96)	(0.93-0.94)

Table legend: ^a^ * = p < .05, ** = p < .01, *** = p < .001.

Alongside the effects of the intervention, Table 2 reveals that there were overall differences between the sites in stair usage. Stair climbing was more common at the Poster + Stairwell messages site (62.3% vs. 41.0%; see Figure 4) whereas stair descent was more common at the Poster alone site (71.3% vs. 65.5%). Finally, there were effects of pedestrian traffic on both ascent and descent such that *less* people used the stairs at higher rates of traffic volume.

### Follow-up questionnaire

The follow-up questionnaire was returned by 123 (24.6%) and 165 (13.8%) employees at the sites respectively. This low response rate suggests the data should be treated with some caution despite the reasonable numbers for statistical comparisons; only employees with complete data were included in analyses. While there were no differences between the gender composition of those returning the questionnaire between sites (Poster + Stairwell messages = 53.4% female; Poster alone = 58.6% females), respondents from the Poster + Stairwell messages site were younger than those from the Poster alone site (mean years = 34.0 SE = 1.3 vs. mean years = 42.2 SE = 0.9; t_251_ = 5.17, p < .001).

Respondents reported greater encouragement to use stairs for the Poster + Stairwell messages campaign (mean = 3.58, SE = 0.15) than for Posters alone (mean = 3.08, SE = 0.12: t_251_ = 2.31, p = .02). Intentions to use the stairs in the future were positive overall, with no differences between sites (Poster + Stairwell messages mean = 3.97, SE = 0.16, Posters alone mean = 3.82, SE = 0.13: t_251_ = 0.75, p = .46). Table 3 summarises agreement with four messages displayed in the stairwell at the Poster + Stairwell messages site, as well as the unused comparison messages, and the results of Bonferroni corrected t-tests. For the messages displayed at the Poster + Stairwell messages site, Table 3a shows no differences between the worksites for agreement with the ‘daily exercise’ or ‘helping weight control’ messages. In contrast, agreement with both calorific expenditure messages were higher at the Poster + Stairwell messages site than at the site not displaying them. As shown in Table 3b, there were no significant differences between the worksites in agreement with statements about stair climbing that were not presented in the stairwell.

**Table 3:** Agreement ratings (SE) at the two worksites for messages in the follow-up questionnaire

	**Poster + Stairwell messages (n = 103)**	**Poster alone (n = 150)**
a) Messages in Stairwell		
Provides daily exercise	5.10(0.10)	4.97(0.09)
Helps control my weight	4.26(0.13)	4.24(0.11)
Burns more calories per minute than jogging	4.35(0.14)	3.85*^a^(0.11)
Burns more calories per minute than rowing	3.69(0.15)	3.16*(0.11)
b) Comparison messages not used in the campaign		
Will keep me fit	4.89(0.10)	5.05(0.08)
Will reduce my risk of a heart attack	4.58(0.11)	4.71(0.10)
Can reduce my cholesterol levels	4.13(0.11)	3.93(0.10)
Will protect me from osteoporosis (brittle bones)	3.91(0.13)	4.15(0.10)

a) messages used in the stairwell at the Poster + Stairwell messages site and b) messages not used in the stairwell at either site. ^a^ Poster + Stairwell messages site > Poster alone site: * = p < .05, ** = p < .01, *** = p < .001.

Finally, exploratory analysis of intentions to use the stairs more in the future used the predictor variables of motivation by the campaign, agreement with the four additional stairwell messages contained in the questionnaire and the interaction terms reflecting potentially better agreement with these measures at the Poster + Stairwell message site which displayed them. All data were mean centred prior to analysis [21]. To control for multi-collinearity of the predictor variables, particularly the stairwell statements, stepwise regression was employed with age, gender and worksite forced into the model. Table 4 summarises the final model accounting for 41.9% of the variance of future intentions to use the stairs more (F_5,247_ = 36.89 p < .001). Gender, encouragement by the campaign and greater agreement that regular stair climbing would help weight control at the Poster + Stairwell messages site relative to the Poster alone site contributed to intention. A model with just the two campaign contributors accounted for 40.1% of the variance of intentions. In follow-up analysis, females (mean = 4.17 SE = 0.15) had more positive intentions than males (mean = 3.69 SE = 0.13: t_251_ = 2.44, p = .02) but did not differ on any of the other variables (all prob. >.12). 

**Table 4:** Predictors of intentions to use the stairs more in the future

**Variable**	**Coefficient**	**SE**	**Standardised coefficient**
Worksite	0.28	0.17	0.09
Age	−0.01	0.01	−0.08
Gender (F > M)	0.38	0.16	0.12* ^a^
Encouraged by campaign	0.65	0.05	0.61***
Helps weight control (interaction with site)	0.11	0.04	0.13**

Table legend: ^a^ * = p < .05, ** = p < .01, *** = p < .001

## **Discussion**

### Calorific messages

Ratings of agreement in pre-testing were uniformly low for the overall weight control message or the specific calorific comparisons with jogging and rowing. If respondents in the previous pre-testing did not believe the messages, they would be unlikely to report them as motivating [19]. Here, where participants were told that the consequences were true, potential motivational effects were enhanced. These findings suggest that campaigns targeting calorific expenditure are feasible, provided the messages convince the audience.

When using quasi-experimental designs, triangulation on the reported outcome is helpful. Analysis of the follow-up questionnaire suggested that messages in stairwells at the Poster + Stairwell messages site *had* affected attitudes. Greater agreement with the calorific statements at the site with supplementary messages, relative to the Poster alone site, echoed the greater increase in stair climbing. The absence of differences between the sites for the messages not displayed at either, makes it unlikely that these differences in attitude reflect more positive overall views of stair climbing at the Poster + Stairwell messages site. It should be noted that employees at both sites could deduce that ‘Regular stair climbing helps control your weight’ on the basis of the main message. While there were no differences in intention between the sites, reported motivation by the main campaign message, and greater agreement that stairs would help weight control at the Poster + Stairwell messages site accounted for 40.1% of the variance of intentions. These self-report data are consistent with objectively measured increases in stair climbing from the campaign. While the relatively low rates of response to the questionnaire prompt some caution, effects of this campaign on attitude and intention has been replicated (Olander & Eves, unpublished observations). As perceived control also contributes to intention and behaviour in the TPB [14], effects of stairwell messages targeting this variable, e.g., stair climbing is easy, would be informative.

### Effects of the intervention

One point should be made explicit before discussing the intervention effects. With only two sites, a comparison of effects *could* reflect structural differences between sites; two lifts at the Poster alone site contrasted with one at the Poster + Stairwell messages site. More available lifts, i.e., Poster alone site, biases behaviour towards their use [7], offering an alternative explanation for differences in response to the campaign. Here though, stair descent was *more* common at the Poster alone site, i.e. the one with more available lifts. Clearly, there were no *overall* biases away from stair use at the Poster alone site to provide a simple confound to explain differential effects of the two interventions.

The core campaign of the poster with the extended message, produced comparable effects to other campaigns using messages that detailed the *amount* of stair climbing required for health benefits [18,22]; the sample-size weighted OR for stair climbing in these previous campaigns (OR = 1.18) is similar to the OR of 1.24 here. The core campaign augmented by stairwell messages had even greater effects (OR = 1.52), comparing favorably with the sample size weighted OR in our previous research and that of others. Further, the effects on stair climbing of a similar campaign targeting heart health which also included stairwell messages were smaller than the current campaign (OR = 1.18 [18]). These comparisons suggest that calorific expenditure may be a particularly fruitful theme for worksite interventions. While weight status of the respondents was not assessed here (see below), increased calorific expenditure may be a general goal of the population.

Two further points from the data merit comment. First, no campaign effects occurred for stair descent, unlike a previous multi-component heart health campaign [18]. It is possible that calorific expenditure is more easily linked to stair climbing in lay consciousness though there is no evidence for this suggestion. Second, effects of traffic in the worksite were the opposite of those seen in public access settings; increased traffic was associated with reduced stair use whereas elevated traffic consistently increases stair climbing in public access settings [23-25]. At work, an employee arriving at the lift may find a colleague already waiting and hence assume the lift would be quicker than the stairs. Any social interaction with the waiting colleague would also bias choice towards the lift. Further, if employees reached the lift together, choice could be constrained by any accompanying colleague who was unwilling or unable to take the stairs. All three of these situations would reduce the number of individuals choosing the stairs consistent with the negative effects of pedestrian traffic on stair use for both ascent and descent.

### Implications

Stair climbing is a vigorous physical activity, with beneficial effects on cardio-respiratory fitness and lipoprotein profiles [26,27]. Further, a recent worksite study [18] revealed greater effects on stair climbing in the overweight (+5.1%; OR = 1.33 vs. +2.5%; OR = 1.12). Similarly, a recent test of a multi-component intervention with a calorie theme in a public access setting also revealed greater effects in overweight commuters [25]. One explanation of these effects is that some overweight individuals may intend to change but feel powerless to achieve change with conventional exercise. Point-of-choice prompts help translate these positive intentions into behaviour. Unlike sport, stair climbing is a physical activity for all. It is not competitive or dependent on sporting ability, requires no equipment, appropriate clothing or exercise partners and can even be performed without others knowing that one is exercising. Stair climbing interventions are six times more cost-effective than their nearest rival [28]. Unlike public access settings, worksites provide an individual with opportunities for stair climbing throughout the day. While stair climbing alone will not reduce obesity, the specificity of its effects to the overweight suggests it is a useful additional tool for public health. An 80 kg man climbing the extra 10 flights daily proposed by this campaign would accumulate physical activity equivalent to three pounds of fat each year, a helpful addition.

### Limitations

The research reported here did not assess weight status but rather the efficacy of a new calorific expenditure campaign for worksites. Hence, testing effects of calorific expenditure campaigns on groups differing in weight status, and *actual* weight, would be informative. Additionally, there was no control site with only point-of-choice prompts. Nonetheless, the sparse evidence for successful increases specific to stair climbing suggests the new approach would be superior to a control intervention; only two out of nine previous studies using a point-of-choice prompt alone [10] report success [29,30]. Further, a 3-week test of the new approach means longer term effects are unknown. Indeed, some dissipation of effects by week three at the Poster + Stairwell messages site occurred. Testing the long term effects of successful worksite site campaigns, and maintenance of any effects after campaign removal, are urgent priorities [10]. Finally, questionnaire measures taken only after the campaign means that attitudes and intentions before the campaign were not partialled out.

## **Conclusions**

Pre-testing in this study indicated that calorific expenditure was a feasible theme for stair climbing interventions if the audience could be convinced by the messages. An extended campaign message outlining the calorific benefits, coupled with a conventional point-of-choice prompt, increased stair climbing by +7.2%. Addition of messages in the stairwell to this core campaign produced a larger increase (+12.3%). Given the equivocal evidence for effectiveness of point-of-choice prompts alone on stair climbing in worksites [7-10], this study suggests multi-component interventions that target attitudes, as well as behaviour at the time choice is made, can substantially increase stair climbing at work.

## **Competing interest**

The authors declare that they have no competing interests.

## **Authors’ contributions**

FFE led the design the study, performed the analyses, and wrote the initial draft. OJW helped in the design of the study, collected the data and commented on drafts. CG helped in the initial design of the project, facilitated access to the worksites and commented on drafts. JC contributed to the design of the project, facilitated access to the worksites and commented on drafts. All authors read and approved the final manuscript.

## Pre-publication history

The pre-publication history for this paper can be accessed here:

http://www.biomedcentral.com/1471-2458/12/423/prepub
